# Rosmarinic Acid Ameliorates Obesity-Associated Metabolic Disturbances and Hepatic Steatosis in Mice with High-Fat Diet-Induced Obesity

**DOI:** 10.3390/ijms27146530

**Published:** 2026-07-22

**Authors:** Mi-Ock Baek, Young-Mo Yang, Eun-Young Kwon, Ji-Young Choi

**Affiliations:** 1 Institute of Well-Aging Medicare & CSU G-LAMP Project Group, Chosun University, Gwangju 61452, Republic of Korea; mobaek@chosun.ac.kr; 2 Department of Pharmacy, College of Pharmacy, Chosun University, Gwangju 61452, Republic of Korea; yyang@chosun.ac.kr; 3 Department of Food Sciences and Nutrition, Kyungpook National University, Daegu 41566, Republic of Korea; eykwon@knu.ac.kr; 4 Department of Food and Nutrition, College of Natural Science and Public Health and Safety, Chosun University, Gwangju 61452, Republic of Korea

**Keywords:** rosmarinic acid, obesity, high-fat diet, hepatic steatosis, lipid metabolism, glucose metabolism

## Abstract

Obesity and obesity-associated hepatic steatosis represent major metabolic health challenges, yet effective pharmacological interventions remain limited. Rosmarinic acid (RA), a natural polyphenol, has been reported to exert anti-obesity effects; however, its specific roles in restoring hepatic lipid homeostasis and modulating glucose metabolism under diet-induced obesity remain unclear. In this study, we investigated the metabolic effects and underlying mechanisms of RA in mice with high-fat diet (HFD)-induced obesity. RA significantly reduced body weight gain and adipose tissue mass without altering total energy intake, accompanied by increased nocturnal energy expenditure and fecal lipid excretion. RA restored hepatic lipid homeostasis by improving circulating lipid profiles and markedly attenuating hepatic steatosis, fibrosis, and hepatocellular injury. These effects were associated with increased fecal lipid excretion, suppression of hepatic lipogenesis, and enhancement of fatty acid oxidation-related markers. Furthermore, RA reduced fasting blood glucose levels and modulated the expression of hepatic glucose metabolism-related genes. Pancreatic immunohistochemistry showed morphological changes in insulin-positive and glucagon-positive cells following RA supplementation. Collectively, these findings indicate that RA ameliorates obesity-associated metabolic disturbances and hepatic steatosis through coordinated regulation of lipid metabolism and hepatic glucose metabolism-related pathways, highlighting its potential relevance for obesity-associated fatty liver conditions.

## 1. Introduction

Metabolic dysfunction-associated steatotic liver disease (MASLD) is a common chronic liver disease worldwide [1]. It is closely associated with obesity, insulin resistance, and dyslipidemia and is characterized by hepatic fat accumulation in the absence of significant alcohol consumption [2,3]. The global prevalence of MASLD is increasing in parallel with the obesity epidemic [1,4], which in turn increases the risk of type 2 diabetes mellitus, cardiovascular disease, and liver-related morbidity and mortality [5,6]. Despite its clinical significance, there is currently no approved pharmacological treatment for MASLD [7]. This emphasizes an urgent need to develop therapeutic strategies that address multiple aspects of metabolic dysfunction.

Recently, there has been an increasing interest in naturally derived polyphenolic compounds as potential agents for the treatment of metabolic diseases, mainly due to their multi-targeted actions, including antioxidant, anti-inflammatory, and metabolic regulatory effects [8,9]. Among these, rosmarinic acid (RA), a phenolic compound highly abundant in rosemary, lemon balm, and perilla, has demonstrated promising anti-obesity and hepatoprotective effects in experimental models [10,11]. Previous studies have shown that RA regulates lipid metabolism by reducing adipocyte hypertrophy and improving lipolysis [12], as well as mitigates systemic inflammation and enhances insulin sensitivity [13,14].

However, the integrated effects of RA on hepatic steatosis, adipose tissue remodeling, and glucose metabolism under high-fat diet (HFD)-induced obesity remain unknown. Therefore, we investigated the metabolic effects of RA supplementation in a mouse model of HFD-induced obesity, with particular attention to energy balance, lipid metabolism, hepatic steatosis, and fasting glucose-related metabolic changes. We further examined the expression of genes and proteins involved in lipogenesis, fatty acid oxidation, and hepatic glucose metabolism to explore the molecular changes associated with RA supplementation.

Our findings demonstrate that RA attenuates obesity-associated hepatic steatosis and metabolic disturbances by regulating lipid metabolism and hepatic glucose metabolism-related pathways in HFD-fed mice, supporting its potential relevance for obesity-associated metabolic disorders.

## 2. Results

### 2.1. RA Supplementation Reduced Body Weight and Adipose Tissue Mass by Enhancing Energy Expenditure and Increasing Fecal Lipid Excretion in HFD-Fed Mice

**Figure 1:**
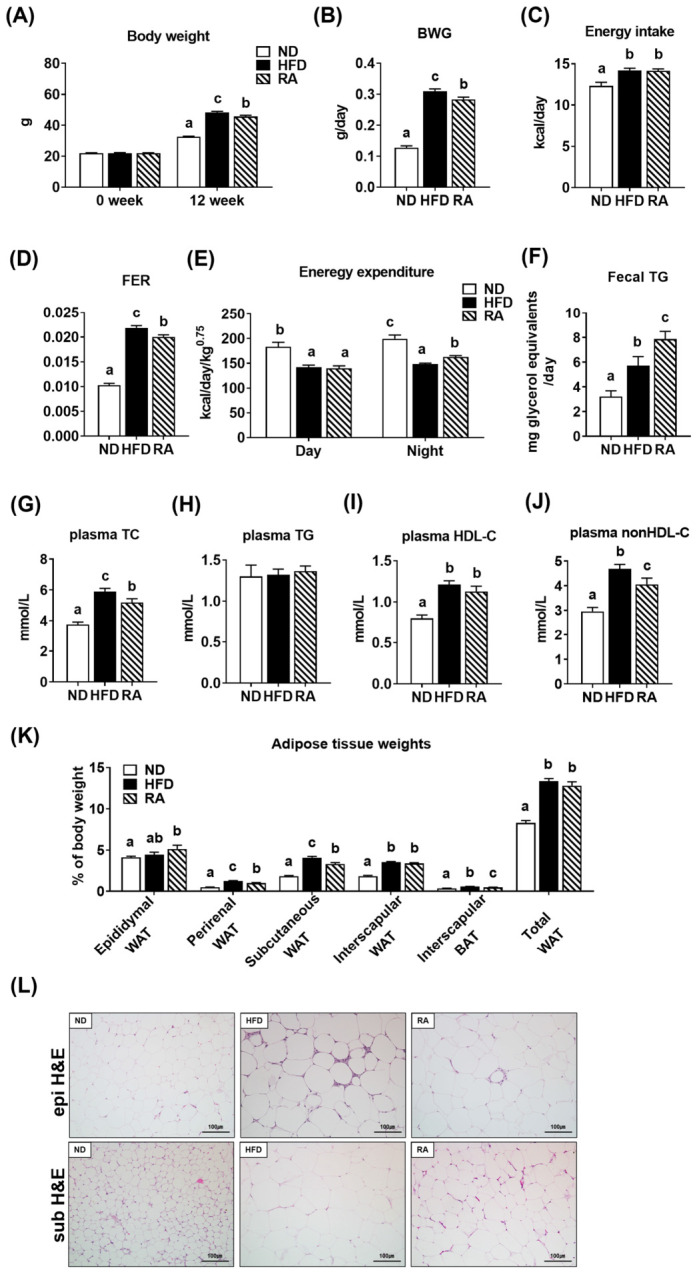
Rosmarinic acid supplementation improved body weight, energy expenditure, and lipid metabolism in HFD-fed mice. (**A**) Body weight, (**B**) body weight gain (BWG), (**C**) total energy intake, and (**D**) food efficiency ratio (FER) after 12 weeks. (**E**) Energy expenditure during the day and night periods assessed by indirect calorimetry. (**F**) Fecal triglyceride (TG) excretion expressed as mg glycerol equivalents/day. (**G**) Plasma total cholesterol (TC). (**H**) Plasma triglyceride (TG). (**I**) Plasma high-density lipoprotein cholesterol (HDL-C) levels. (**J**) Plasma non-HDL cholesterol levels. (**K**) Relative weights of adipose tissues expressed as percentages of body weight, including epididymal, perirenal, subcutaneous, and interscapular white adipose tissue (WAT), total WAT, and interscapular brown adipose tissue (BAT). (**L**) Histological analysis of epididymal and subcutaneous WAT stained with hematoxylin and eosin (H&E) (scale bar: 100 μm). Data are presented as mean ± SEM (*n* = 6 per group). Statistical significance was assessed using one-way ANOVA followed by Tukey’s post hoc test. For variables measured at multiple time points, group differences were analyzed separately at each time point using one-way ANOVA followed by Tukey’s post hoc test. ^abc^ Means not sharing a common letter are significantly different among the groups (*p* < 0.05). ND, normal diet (AIN-93G); HFD, high-fat diet (60 kcal% fat); RA, HFD + rosmarinic acid (0.0318%).

To investigate the effect of RA on the progression of obesity, we first examined body weight changes in mice fed an HFD for 12 weeks. HFD-fed mice exhibited considerably higher body weight and body weight gain (BWG) compared with those fed a normal diet (ND). However, RA markedly attenuated both body weight and BWG in HFD-fed mice (Figure 1A,B). To determine whether these effects were due to altered energy intake, total food consumption was measured. No significant differences were observed between the HFD and RA groups (Figure 1C). However, the food efficiency ratio (FER) was significantly lower in the RA-supplemented groups than in the HFD group (Figure 1D). These findings suggest that RA mitigates weight gain independent of reduction in energy intake. Indirect calorimetry was used for metabolic assessment. HFD-fed mice showed reduced energy expenditure compared with ND-fed mice, particularly during the dark phase. Conversely, RA supplementation significantly increased nocturnal energy expenditure compared with the HFD group, indicating enhanced metabolic activity (Figure 1E). Fecal lipid analysis showed that HFD increased fecal triglyceride excretion, which was further increased by RA supplementation (Figure 1F). This indicates that RA enhances lipid excretion and/or inhibits intestinal lipid absorption, thereby reducing systemic lipid accumulation.

Regarding lipid metabolism, RA significantly reversed HFD-induced elevated plasma total cholesterol (TC) (Figure 1G), whereas plasma triglyceride (TG) levels did not show significant alterations (Figure 1H). Plasma HDL-cholesterol (HDL-C) levels were higher in the HFD group than in the ND group, and there was no significant difference between the RA and HFD groups (Figure 1I). In contrast, RA significantly reduced non-HDL cholesterol levels compared with the HFD group (Figure 1J). As shown in Figure 1K, HFD increased the relative weights of several adipose depots when expressed as percentages of body weight. RA significantly reduced the relative weights of perirenal and subcutaneous WAT compared with the HFD group. In contrast, the relative weights of epididymal WAT, interscapular WAT, and total WAT did not significantly differ between the HFD and RA groups. The relative weight of interscapular BAT was also significantly altered by RA supplementation. The absolute adipose tissue weights are provided in Supplementary Figure S3A. Histological analysis showed that adipocytes in the epididymal and subcutaneous WAT of HFD-fed mice were considerably less hypertrophied in the RA-treated groups (Figure 1L). These findings suggest that RA not only reduces adipose tissue mass but also improves adipocyte morphology. Thus, RA effectively attenuates diet-induced obesity by enhancing energy expenditure, improving blood and fecal lipid profiles, reducing adipose tissue accumulation, and alleviating adipocyte hypertrophy, independent of energy intake.

### 2.2. RA Supplementation Improved Plasma Lipid Profiles and Adipokine Levels in HFD-Fed Mice

**Figure 2:**
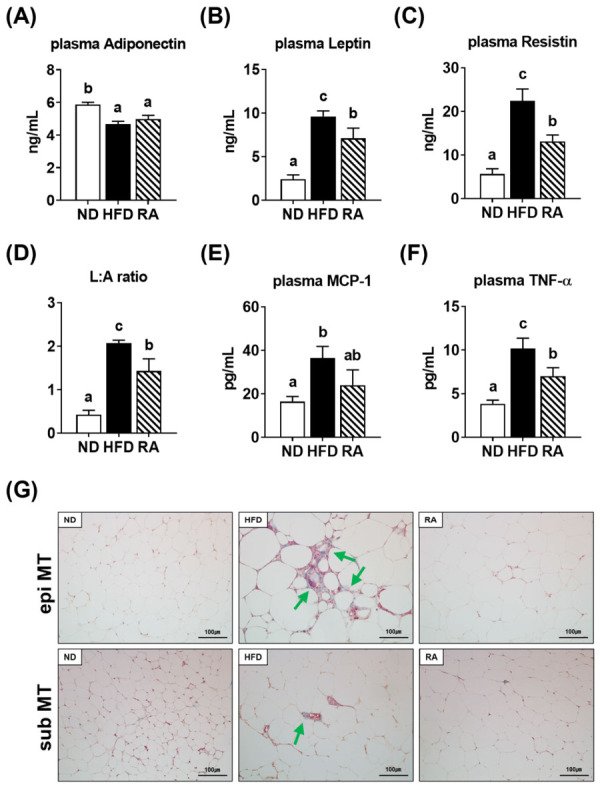
Rosmarinic acid supplementation improved adipokine profiles, reduced systemic inflammation, and attenuated adipose tissue fibrosis in HFD-fed mice. (**A**) Plasma adiponectin, (**B**) leptin, (**C**) and resistin levels, and (**D**) leptin-to-adiponectin ratio (L:A ratio). (**E**,**F**) Plasma levels of the pro-inflammatory cytokines MCP-1 and TNF-α. (**G**) Masson’s trichrome staining of epididymal and subcutaneous WAT showing fibrosis; green arrows indicate areas of collagen-positive areas (scale bar: 100 μm). Data are presented as mean ± SEM (*n* = 6 per group). Statistical significance was assessed using one-way ANOVA followed by Tukey’s post hoc test. ^abc^ Means not sharing a common letter are significantly different among the groups (*p* < 0.05). ND, normal diet (AIN-93G); HFD, high-fat diet (60 kcal% fat); RA, HFD + rosmarinic acid (0.0318%).

Because adipokine imbalance plays a critical role in obesity-related metabolic disorders, we examined whether RA could restore circulating adipokine profiles in HFD-fed mice. Compared with the ND group, HFD significantly decreased plasma adiponectin levels while increasing leptin and resistin concentrations. The leptin-to-adiponectin (L:A) ratio was also markedly elevated in HFD-fed mice (Figure 2A–D). RA supplementation did not significantly alter adiponectin levels but significantly reduced leptin and resistin levels, as well as the L:A ratio, compared with the HFD group. These findings suggest that RA may alleviate adipokine imbalance and support metabolic homeostasis under obesogenic conditions.

Next, we evaluated systemic inflammation by measuring the levels of pro-inflammatory cytokines. Plasma levels of MCP-1 and TNF-α were significantly increased in the HFD group, while RA supplementation significantly reduced TNF-α levels and showed a decreasing trend in MCP-1 compared with the HFD group (Figure 2E,F). These data demonstrate the anti-inflammatory potential of RA in modulating adipose tissue inflammation in diet-induced obesity. Histological analysis of epididymal and subcutaneous WAT using Masson’s trichrome staining revealed notable collagen deposition (fibrosis) in the HFD group. In contrast, the RA-supplemented groups showed significantly fewer fibrotic areas, suggesting that RA may alleviate adipose tissue remodeling and fibrosis associated with chronic inflammation (Figure 2G). Together, these data provide evidence that RA supplementation improves circulating adipokine levels and lowers inflammatory cytokine generation while preventing adipose tissue fibrosis, thereby contributing to the metabolic benefits observed in diet-induced obesity.

### 2.3. RA Supplementation Alleviated Hepatic Steatosis and Liver Injury-Related Markers in HFD-Fed Mice

**Figure 3:**
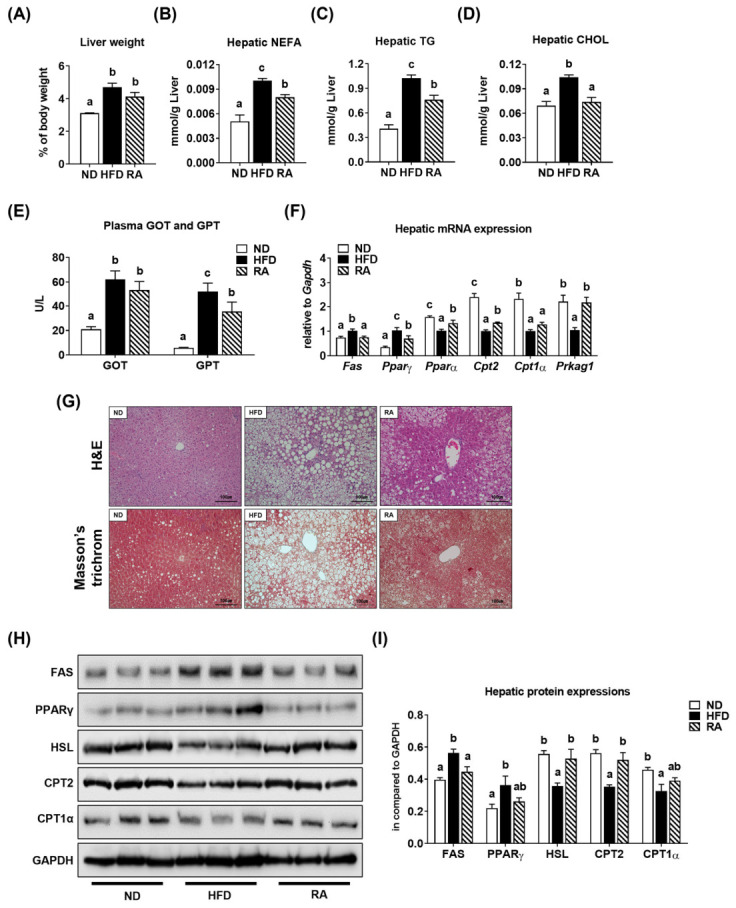
Rosmarinic acid ameliorated obesity-associated hepatic steatosis and fibrosis by regulating lipid metabolism and enhancing fatty acid oxidation in HFD-fed mice. (**A**) Relative liver weight expressed as percentage of body weight, (**B**) hepatic non-esterified fatty acid (NEFA), (**C**) hepatic triglyceride (TG), and (**D**) hepatic total cholesterol (CHOL) contents. (**E**) Plasma liver enzymes GOT and GPT, expressed as U/L. (**F**) Hepatic mRNA expression of *Fas*, *Pparγ*, *Pparα*, *Cpt2*, *Cpt1α*, and *Prkag1*. (**G**) Representative hepatic histology: Hematoxylin and eosin (H&E) staining showing hepatic steatosis, and Masson’s trichrome staining showing collagen deposition (fibrosis) (scale bar: 100 μm). (**H**) Representative Western blot images of FAS, PPARγ, HSL, CPT2, CPT1α and GAPDH. The expected molecular weights of the target proteins are shown in Supplementary Figure S1. (**I**) Quantification of hepatic protein expression normalized to GAPDH. Data are presented as mean ± SEM (*n* = 6 per group). Statistical significance was assessed using one-way ANOVA followed by Tukey’s post hoc test. ^abc^ Means not sharing a common letter are significantly different among the groups (*p* < 0.05). ND, normal diet (AIN-93G); HFD, high-fat diet (60 kcal% fat); RA, HFD + rosmarinic acid (0.0318%).

To investigate the effect of RA on hepatic lipid accumulation, we measured liver weight and hepatic lipid content. HFD significantly increased relative liver weight, expressed as a percentage of body weight, as well as hepatic non-esterified fatty acid (NEFA), triglycerides (TG), and total cholesterol (CHOL) compared with the ND group. The relative liver weight did not significantly differ between the HFD and RA groups. However, RA significantly reduced hepatic NEFA and TG levels compared with the HFD group, whereas hepatic CHOL levels were not significantly different between the ND and RA groups (Figure 3A–D). The absolute liver weight is provided in Supplementary Figure S3B. To further investigate hepatic function, we measured the plasma levels of liver enzymes, including glutamate oxaloacetate transaminase (GOT) and glutamate pyruvate transaminase (GPT). HFD-fed mice exhibited increased plasma GOT and GPT activities, indicative of hepatocellular injury. RA significantly reduced plasma GPT activity, whereas plasma GOT activity did not significantly differ between the HFD and RA groups (Figure 3E). Histological analysis also supported these results. Hematoxylin and eosin (H&E) staining revealed that the HFD group exhibited severe hepatic steatosis, characterized by numerous macrovesicular lipid droplets and disrupted hepatocellular structure. However, RA supplementation markedly reduced both the size and number of lipid droplets, and hepatic structure remained more intact. Masson’s trichrome staining showed diet-associated histological alterations in liver tissue, which appeared less pronounced in the RA-treated group (Figure 3G). Given the absence of fibrosis-specific quantitative assessments, these histological findings were not interpreted as definitive evidence of hepatic fibrosis or antifibrotic activity. At the molecular level, RA regulated the expression of key genes involved in lipid metabolism. Hepatic mRNA levels of the lipogenic markers *Fas* and *Pparγ* were significantly elevated in the HFD group but were notably suppressed by RA. In contrast, the RA-treated group showed significantly higher expression of genes associated with fatty acid β-oxidation, including *Pparα*, *Cpt2*, and *Cpt1α*. Moreover, RA supplementation increased *Prkag1* expression, which encodes the AMPKγ1 subunit (Figure 3F). Western blot analysis showed similar changes at the protein level, with reduced FAS and PPARγ expression and increased HSL, CPT2, and CPT1α expression in the RA group compared with the HFD group (Figure 3H,I). These findings indicate that RA attenuates obesity-associated hepatic steatosis by reducing hepatic lipid synthesis, enhancing fatty acid oxidation, and preserving hepatic structural integrity, thereby alleviating diet-induced hepatic steatosis and fibrosis.

### 2.4. RA Supplementation Improved Fasting Blood Glucose Levels and Regulated Hepatic Glucose Metabolism-Related Genes in HFD-Fed Mice

**Figure 4:**
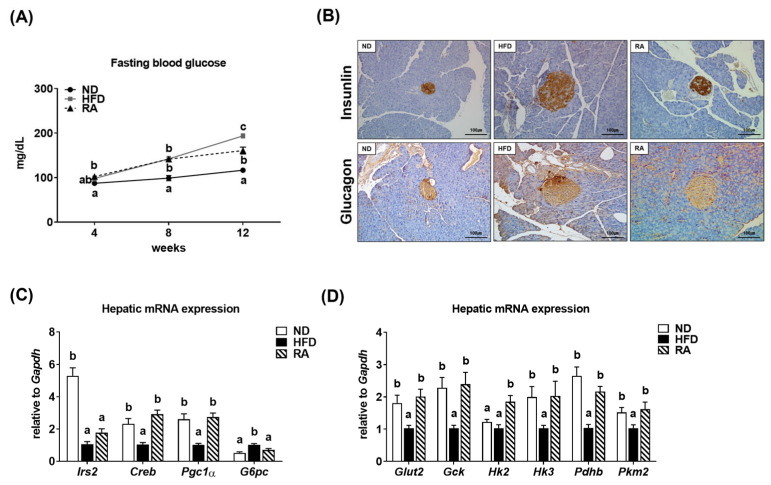
Rosmarinic acid reduced fasting blood glucose levels and modulated pancreatic endocrine cell morphology and hepatic glucose metabolism-related gene expression in HFD-fed mice. (**A**) Fasting blood glucose levels measured at weeks 4, 8, and 12. (**B**) Immunohistochemical staining of pancreatic insulin and glucagon. Brown staining indicates positive immunoreativity (scale bar: 100 μm). (**C**) Hepatic mRNA expression of *Irs2*, *Creb*, *Pgc1α*, and *G6pc*. (**D**) Hepatic mRNA expression of *Glut2*, *Gck*, *Hk2*, *Hk3*, *Pdhb*, and *Pkm2*. Data are presented as mean ± SEM (*n* = 6 per group). Statistical significance was assessed using one-way ANOVA followed by Tukey’s post hoc test. For variables measured at multiple time points, group differences were analyzed separately at each time point using one-way ANOVA followed by Tukey’s post hoc test. ^abc^ Means not sharing a common letter are significantly different among the groups (*p* < 0.05). ND, normal diet (AIN-93G); HFD, high-fat diet (60 kcal% fat); RA, HFD + rosmarinic acid (0.0318%).

To examine the effects of RA on glucose-related metabolic parameters under obese conditions, we measured fasting blood glucose levels. Mice in the HFD group showed increased fasting blood glucose levels for 12 weeks compared with those in the ND group. In contrast, RA supplementation significantly reduced fasting blood glucose levels at week 12 compared with the HFD group (Figure 4A).

Morphological analysis using immunohistochemical (IHC) staining showed changes in insulin-positive β-cells and glucagon-positive α-cells in HFD-fed mice. RA supplementation attenuated these HFD-induced morphological changes in pancreatic endocrine cells (Figure 4B).

To examine whether RA affected hepatic glucose metabolism-related gene expression, we analyzed the mRNA expression of genes associated with insulin signaling-related pathways, gluconeogenesis, glucose transport, glycolysis, and pyruvate metabolism. RA restored the HFD-induced changes in *Irs2*, *Creb*, and *Pgc1α* expression and reduced the expression of *G6pc*, a key gluconeogenic enzyme. In addition, RA modulated the expression of genes associated with glucose transport, glycolysis, and pyruvate metabolism, including *Glut2*, *Gck*, *Hk2*, *Hk3*, *Pdhb*, and *Pkm2* (Figure 4C,D). These findings suggest that RA reduced fasting blood glucose levels and influenced hepatic glucose metabolism-related gene expression in HFD-fed mice.

## 3. Discussion

Obesity and obesity-associated hepatic steatosis have emerged as major health challenges, driven by modern dietary patterns and insufficient physical activity [15,16]. However, effective therapeutic options remain limited. In this study, we investigated how RA, a natural polyphenol compound, modulates metabolic alterations in a mouse model of HFD-induced obesity [17,18]. The results suggest that RA exerts beneficial effects on body weight regulation, lipid metabolism, hepatic steatosis, and fibrosis within this obesity-associated metabolic context.

A notable finding of this study was that RA significantly reduced body weight and adipose tissue mass in HFD-fed mice without altering total energy intake, suggesting that RA’s anti-obesity effect is not attributable to appetite suppression. However, the RA-supplemented group exhibited a significant increase in nocturnal energy expenditure and fecal lipid excretion, suggesting increased energy expenditure and fecal lipid elimination [19]. The increased fecal triglyceride excretion observed in the RA-supplemented group suggests that RA may reduce intestinal lipid absorption and/or enhance fecal lipid elimination. Therefore, the body weight-lowering and hepatic lipid-lowering effects of RA may not be explained solely by the direct regulation of hepatic lipid metabolism. Instead, reduced intestinal lipid availability resulting from increased fecal lipid loss may have contributed to the decrease in systemic lipid burden, adipose tissue accumulation, and hepatic triglyceride deposition. This interpretation provides an additional mechanism by which RA may protect against HFD-induced metabolic and hepatic alterations. In addition, the improvement in the FER supports a shift in metabolism toward more efficient energy utilization rather than energy storage [20].

RA supplementation also improved several systemic lipid parameters. RA significantly reduced plasma TC and non-HDL cholesterol compared with the HFD group, had no effect on plasma TG, and slightly reduced HDL cholesterol compared with the HFD group. Although hepatic triglyceride content was significantly reduced by RA, plasma triglyceride levels were not significantly altered. This apparent discrepancy may be explained by the dynamic regulation of circulating triglycerides, which are influenced by hepatic VLDL-triglyceride secretion, peripheral triglyceride clearance, intestinal lipid absorption, and adipose tissue lipid mobilization. In addition, plasma ApoB levels were not significantly different among the ND, HFD, and RA groups (Figure S2), indicating that RA supplementation did not increase circulating ApoB levels. Therefore, the unchanged plasma triglyceride level was unlikely to be explained by a marked compensatory increase in ApoB-containing lipoproteins. However, because VLDL secretion and triglyceride clearance were not directly measured, further studies assessing VLDL secretion and triglyceride clearance will help clarify this mechanism.

These changes in lipid profiles suggest a reduced risk of cardiovascular complications associated with dyslipidemia [21,22,23]. These effects are likely associated with increased lipid mobilization and mitochondrial fatty acid oxidation [24]. Molecular analyses in the liver showed that RA downregulated genes (*Fas*, *Pparγ*) and proteins (FAS, PPARγ) associated with lipid synthesis and upregulated genes and proteins (*Pparα*, *Cpt1α*, *Cpt2*) and lipolytic factor (HSL) involved in β-oxidation of fatty acids [25,26]. This change in transcriptional regulation promotes liver lipid metabolism, leading to a transition from lipid synthesis and storage to lipid degradation and utilization, thereby alleviating lipid accumulation in the liver. RA also increased hepatic *Prkag1* mRNA expression, which encodes the AMPKγ1 subunit. However, because AMPK phosphorylation and ACC phosphorylation were not measured, this finding cannot be interpreted as direct evidence of AMPK pathway activation. Further studies are required to determine whether AMPK signaling contributes to the hepatic lipid-lowering effects of RA.

In addition to lipid metabolism, RA improved adipokine balance and reduced systemic inflammation, both of which are critical for metabolic dysfunction associated with obesity [27]. Insulin resistance and chronic inflammation are often exacerbated by HFD feeding [28], which typically results in decreased plasma adiponectin and increased leptin and resistin levels [29,30]. Although RA supplementation did not affect adiponectin levels, it effectively reversed this imbalance by reducing leptin and resistin levels and thereby lowering the L:A ratio. Additionally, RA considerably reduced plasma TNF-α levels and showed a decreasing trend in MCP-1 levels. These anti-inflammatory effects were confirmed through histological investigation, revealing reduction in collagen deposition (fibrosis) in epididymal and subcutaneous WAT. Adipose tissue fibrosis, a hallmark of chronic inflammation in obesity, interferes with normal function of adipocytes and promotes insulin resistance [31,32]. Reduction in inflammation and fibrosis by RA may contribute to improved adipose tissue function and metabolic homeostasis.

One of the most important findings of this study was that RA attenuated obesity-associated hepatic steatosis and related liver injury markers. Mice fed an HFD exhibited increased relative liver weight and elevated hepatic FA, TG, and CHOL levels. Furthermore, HFD-fed mice showed elevated plasma GOT and GPT activities, indicative of hepatocellular injury [33]. Although relative liver weight and plasma GOT activity did not significantly differ between the HFD and RA groups, RA supplementation reduced hepatic FA and TG levels and lowered plasma GPT activity. These findings support the hepatoprotective potential of RA against lipid accumulation-associated liver damage. Histological analyses further showed that RA treatment markedly attenuated hepatic steatosis and improved liver histological alterations. Importantly, this hepatoprotective effect may be partly linked to the increase in fecal triglyceride excretion. By promoting fecal lipid loss or limiting intestinal lipid absorption, RA may reduce systemic lipid availability, thereby contributing to the attenuation of hepatic triglyceride accumulation and steatosis. Collectively, these findings suggest that RA has hepatoprotective potential against hepatic steatosis in an obesity-associated metabolic setting.

RA also affected fasting blood glucose levels and hepatic glucose metabolism-related gene expression. RA supplementation reduced fasting blood glucose levels in HFD-fed mice, suggesting a partial improvement in fasting glycemic status. At the molecular level, RA restored the HFD-induced changes in the expression of genes related to insulin signaling-associated pathways and mitochondrial function, including *Irs2*, *Creb*, and *Pgc1α* [34,35]. RA also reduced the expression of *G6pc*, a key gluconeogenic enzyme involved in hepatic glucose production [36]. RA modulated the expression of genes associated with glucose transport, glycolysis, and pyruvate metabolism, including *Glut2*, *Gck*, *Hk2*, *Hk3*, *Pdhb*, and *Pkm2* [37,38]. These findings suggest that RA may influence hepatic glucose metabolism in HFD-fed mice. However, because GTT, ITT, plasma insulin/HOMA-IR, and AKT phosphorylation were not assessed in this study, the present data do not allow a direct conclusion regarding improved insulin sensitivity. Future studies incorporating these analyses will help clarify whether RA directly improves insulin sensitivity and systemic glucose homeostasis.

This study has several limitations. Although RA reduced fasting blood glucose levels and modulated hepatic glucose metabolism-related gene expression, GTT, ITT, plasma insulin/HOMA-IR, and AKT phosphorylation analyses were not performed. Therefore, the present data do not allow a direct conclusion regarding improved insulin sensitivity. In addition, hepatic gene expression was normalized using *Gapdh* as a single reference gene. Although the raw *Gapdh* Ct values did not differ significantly among the experimental groups, the use of an additional validated housekeeping gene would have strengthened the reliability of the normalization, particularly for genes involved in glucose and glycolytic metabolism. Moreover, although RA increased hepatic *Prkag1* mRNA expression, AMPK phosphorylation and ACC phosphorylation were not assessed; thus, AMPK pathway activation cannot be directly inferred. Pancreatic IHC findings were interpreted as morphological observations because beta-cell mass, C-peptide levels, and insulin secretion were not measured. Furthermore, the RA dose used in this study was not determined based on pharmacokinetic, tissue distribution, or dose–response analyses. Although the estimated daily RA intake was approximately 29.7 mg/kg body weight/day, plasma RA levels and hepatic or adipose tissue RA concentrations were not measured. Therefore, systemic exposure and tissue distribution of RA could not be directly evaluated in the present study. Future studies incorporating these functional and pharmacokinetic analyses will help clarify the mechanisms underlying the metabolic effects of RA.

## 4. Conclusions

In conclusion, this study demonstrates that RA supplementation attenuated HFD-induced hepatic lipid accumulation and metabolic alterations in mice. RA reduced body weight gain and decreased the relative weights of selected adipose depots without decreasing total energy intake, and these effects were accompanied by increased nocturnal energy expenditure and fecal triglyceride excretion. RA also improved circulating lipid profiles and reduced hepatic lipid accumulation and histological steatosis. These effects were associated with the suppression of hepatic lipogenesis-related markers and the enhancement of fatty acid oxidation-related markers. In addition, RA reduced fasting blood glucose levels and modulated the expression of hepatic glucose metabolism-related genes. Although RA increased hepatic *Prkag1* mRNA expression, GTT, ITT, plasma insulin/HOMA-IR, AKT phosphorylation, AMPK phosphorylation, and ACC phosphorylation were not assessed in this study. Therefore, further studies are needed to clarify the direct effects of RA on insulin sensitivity and AMPK signaling. Overall, these findings suggest that RA may protect against HFD-induced hepatic lipid accumulation through coordinated regulation of lipid metabolism, fecal lipid excretion, and hepatic lipid homeostasis.

## 5. Materials and Methods

### 5.1. Animal Experimental Design and Diet

C57BL/6J mice (four-week-old, male, *n* = 6) were obtained from Jackson Laboratories (Bar Harbor, ME, USA) and housed under controlled conditions (22 ± 2 °C, 12-h light/dark cycle, relative humidity 50 ± 10%), with free access to water and food. After one week of adaptation, mice were randomly divided into three groups: normal diet (ND, AIN-93G, *n* = 6), high-fat diet (HFD, *n* = 6, 60 kcal% fat), and HFD supplemented with rosmarinic acid (R4033; Sigma-Aldrich, St. Louis, MO, USA) (0.0318%, *n* = 6). Mice were fed the experimental diets for 12 weeks. RA was incorporated into the HFD at 0.0318% *w*/*w* during diet preparation and thoroughly mixed to ensure homogeneous distribution in the diet. Food intake was monitored daily by measuring the remaining diet, whereas water was provided ad libitum throughout the experimental period but was not quantitatively monitored. The estimated daily intake of RA was calculated using the following formula: RA intake (mg/kg body weight/day) = food intake (g/day) × 0.318 mg RA/g diet ÷ body weight (kg). Based on food intake and body weight during the experimental period, the estimated RA intake was approximately 29.7 mg/kg body weight/day. The detailed compositions of the experimental diets are presented in Table 1. At the end of the experimental period, mice were fasted for 12 h and anesthetized with isoflurane. Blood samples were collected from the inferior vena cava into heparin-coated tubes and centrifuged to obtain plasma. Plasma samples were stored at −80 °C until analysis of plasma lipid profiles, adipokine levels, and hormone concentrations. The liver, muscle, and adipose tissues were dissected, rinsed with physiological saline, weighed, immediately frozen in liquid nitrogen, and stored at −70 °C until further analysis. Animal studies were conducted in accordance with protocols approved by the Kyungpook National University Industry Foundation (Approval No. KNU 2017-0109).

### 5.2. Measurement of Energy Expenditure

Energy expenditure was assessed using an indirect calorimeter (Oxylet; Panlab, Cornella, Spain). Mice were individually housed in metabolic chambers at 25 °C with free access to food and water. The O_2_ and CO_2_ analyzers were calibrated using high-purity gases. Validation of O_2_ consumption (VO_2_) and CO_2_ production (VCO_2_) was monitored at 3-min intervals for 24 h using automated data acquisition software (Chart 5.2; AD Instrument, Sydney, Australia). Energy expenditure (EE) for each mouse was calculated using the following formula [39]:

EE (kcal·day^−1^·bodyweight^−0.75^) = VO_2_ × 1.44 × [3.815 + (1.232 × VCO_2_/VO_2_)

### 5.3. Morphology of Liver and Adipose Tissues

The liver, epididymal white adipose tissue (epiWAT), and subcutaneous white adipose tissue (subWAT) were excised and fixed in a buffer solution containing 10% formalin. Fixed samples were embedded in paraffin, sliced into 4 µm-thick sections, and stained with hematoxylin and eosin (H&E) or Masson’s trichrome (MT). For immunohistochemistry (IHC) staining of pancreatic insulin and glucagon, paraffin-embedded pancreatic sections were incubated with primary antibodies against insulin (β-cells) and glucagon (α-cells), followed by incubation with appropriate secondary antibodies. All stained sections were examined under an optical microscope (Axioscope; Carl Zeiss, Oberkochen, Germany) at 200× magnification.

### 5.4. Biochemical Parameters of Plasma, Hepatic, and Fecal Lipids

Plasma levels of triglycerides (TG), total cholesterol (TC), and high-density lipoprotein cholesterol (HDL-C) were determined using a commercial kit (Asan Pharm Co., Seoul, Republic of Korea). Non-HDL-C levels were calculated as follows: non-HDL-C = TC − HDL-C. Hepatic and fecal lipid contents were calculated according to a previously described method [40]. The dried lipid extracts were dissolved in 1 mL of ethanol, and 200 µL of each sample was emulsified by adding Triton X-100 (Sigma-Aldrich, St. Louis, MO, USA) and sodium cholate solution in distilled water. Hepatic TG and cholesterol contents were measured using the same enzymatic kits used for plasma lipid analysis. Hepatic fatty acid content was measured using a Non-Esterified Fatty Acid (NEFA) assay kit (FUJIFILM Wako Pure Chemical Corporation, Osaka, Japan), and thus the reported values represent non-esterified (free) fatty acids rather than the total fatty acid pool including esterified lipid fractions.

### 5.5. Determination of Hormones and Adipokines in Plasma

Plasma adipokine (leptin and resistin) levels were measured using the MILLIPLEX^®^ MAP mouse metabolic magnetic bead panel (Merck; Rahway, NJ, USA) according to the manufacturer’s protocol. Adiponectin concentrations were quantified using mouse adiponectin/Acrp30 (R&D Systems, Bio-Techne, Minneapolis, MN, USA).

### 5.6. Fasting Blood Glucose Measurement

Fasting blood glucose (FBG) levels were measured from blood samples obtained from the tail vein, collected after a 12-h fasting period, using a glucose analyzer (One Touch Select Plus, LifeScan Inc., Malvern, PA, USA).

### 5.7. Determination of Hepatic Enzyme Activity

The plasma levels of glutamic oxaloacetic transaminase (GOT) and glutamic pyruvic transaminase (GPT) were determined using commercial assay kits (Asan Pharm Co., Seoul, Republic of Korea) following the manufacturer’s instructions. Enzyme activities were calculated using the manufacturer-provided calibration values and expressed as U/L.

### 5.8. Determination of Plasma Cytokine Concentrations

Plasma concentrations of monocyte chemoattractant protein-1 (MCP-1) and tumor necrosis factor-alpha (TNF-α) were measured using the MILLIPLEX^®^ MAP mouse cytokine/chemokine magnetic bead panel (Merck) according to the manufacturer’s instructions.

### 5.9. Quantitative Real-Time PCR (RT-qPCR)

Total RNA was extracted from tissue samples using NucleoZOL reagent (Macherey-Nagel, Düren, Germany) following the manufacturer’s instructions. Total RNA (1 μg) was reverse-transcribed into cDNA using TOPscript RT DryMIX (dT18) (Enzynomics, Daejeon, Republic of Korea). RT-qPCR was performed using TOPreal™ SYBR Green qPCR PreMIX (Enzynomics) on a CFX Connect Real-Time System (Bio-Rad, Hercules, CA, USA). Gene-specific primers for mouse targets are listed in Table 2. Relative gene expression levels were normalized against *Gapdh* using the 2^−△△Ct^ method.

### 5.10. Western Blotting

Liver proteins were extracted using tissue protein extraction reagent (T-PER) buffer (Thermo Fisher Scientific, Rockford, IL, USA) containing protease and phosphatase inhibitor cocktails. Protein concentrations were determined using the Bradford method and subsequently subjected to sodium dodecyl sulfate-polyacrylamide gel electrophoresis (SDS-PAGE) on 10% SDS polyacrylamide gel. The proteins were transferred onto polyvinylidene fluoride membranes (Merck). Membranes were blocked with Tris-buffered saline containing 0.1% Tween-20 (TBST) supplemented with 5% bovine serum albumin (BSA) or 5% skim milk for 1 h, followed by incubation with primary antibodies: rabbit anti-FAS (1:1000; Cell Signaling Technology, Danvers, MA, USA, #3180), anti-PPARγ (peroxisome proliferator-activated receptor gamma) (1:1000; Abcam, Cambridge, UK, ab209350), anti-LIPE (lipase E, hormone-sensitive lipase, HSL) (1:1000; Abcam, ab45422), anti-CPT2 (carnitine palmitoyl transferase 2) (1:1000; Abcam, ab181114), and anti-CPT1A (carnitine palmitoyl-CoA transferase 1-alpha) (1:1000; Abcam, ab128568). Glyceraldehyde-3-phosphate dehydrogenase (GAPDH) was used as a loading control and detected using mouse anti-GAPDH antibody (1:1000; Santa Cruz Biotechnology, Dallas, TX, USA, sc-32233). The membranes were then incubated with horseradish peroxidase (HRP)-conjugated secondary antibodies: rabbit anti-IgG (1:3000; Cell Signaling, #7074S) or mouse anti-IgG (1:3000; Cell Signaling, #7076S). Immunoreactive bands were visualized using the Westar Eta C Ultra 2.0 chemiluminescence detection system (Cyanagen, Bologna, Italy), and band intensities were quantified using ImageJ software (version 1.54t; National Insititutes of Health, Bethesda, MD, USA).

### 5.11. Statistical Analysis

All data are presented as mean ± standard error of the mean (SEM). Statistical analyses were performed using the Statistical Package for Social Sciences (SPSS) software version 23.0 (SPSS Inc., Chicago, IL, USA). Data distribution was assessed using the Shapiro–Wilk normality test prior to statistical comparisons. For comparisons among the three experimental groups, one-way analysis of variance (ANOVA) followed by Tukey’s post hoc test was performed. For variables measured at multiple time points, group differences were analyzed at each time point using one-way ANOVA followed by Tukey’s post hoc test. Statistical significance was set at *p* < 0.05. Different letters indicate significant differences among groups.

## Figures and Tables

**Table 1:** Diet composition for animal experiment.

Ingredient (g)	ND	HFD	RA
Casein	200	265	265
Corn Starch	397.486	0	0
Sucrose	100	90	90
Maltodextrin	132	160	160
Cellulose	50	65.6	65.6
Soybean oil	70	30	30
Lard	0	310	310
Mineral mix ^1^	35	48	48
Vitamin mix ^2^	10	21	21
Calcium phosphate, dibasic	0	3.4	3.4
TBHQ, antioxidant	0.014	0	0
L-Cystine	3	4	4
Cholin bitartrate	2.5	3	3
Rosmarinic acid			0.318
Total (g)	1000	1000	1000.318
Total energy (kcal)	4000	5220	5220

ND, normal diet (AIN-93G); HFD, high-fat diet (60% kcal fat); RA, rosmarinic acid (HFD + 0.0318% RA). ^1^ AIN-93G—Mineral Mix, ^2^ AIN-93G—Vitamin mix.

**Table 2:** Primers used for quantitative real-time PCR.

Primer	Primer Direction	Primer Sequence (5′-3′)
*Gapdh:* Glyceraldehyde-3-phosphate dehydrogenase	Forward Reverse	AAGGTCATCCCAGAGCTGAA CTGCTTCACCACCTTCTTGA
*Creb:* cAMP response element-binding protein	Forward Reverse	GAAGAAGCAGCACGGAAGAGA TCT CTTGCTGCCTCCCTGTT
*Cpt1α*: Carnitine palmitoyl-CoA transferase 1 alpha	Forward Reverse	ATCTGGATGGCTATGGTCAAGGTC GTGCTGTCATGCGTTGGAAGTC
*Cpt2*: Carnitine palmitoyl-CoA transferase 2	Forward Reverse	CAACTCGTATACCCAAACCCAGTC GTTCCCATCTTGATCGAGGACATC
*Fas*: Fatty acid synthase	Forward Reverse	GCTGCGGAAACTTCAGGAAAT AGAGACGTGTCACTCCTGGACTT
*G6pc:* Glucose-6-phosphatase catalytic subunit	Forward Reverse	GGAGGAAGGATG GAGGAAGGAATG GGTCAGCAATCACAGACACAAGG
*Gck:* Glucokinase	Forward Reverse	CAGGACAGTGGAGCGTGAAGAC TTACAGGGAAGGAGAAGGTGAAGC
*Glut2:* Glucose transporter type 2	Forward Reverse	GTCAGAAGACAAGATCACCGGA AGGTGCATTGATCACACCGA
*Hk2:* Hexokinase 2	Forward Reverse	GAGAACCGTGGACTGGACAA CCAGGAAGGACACGTCACAT
*Hk3:* Hexokinase 3	Forward Reverse	GAGAACCGTGGACTGGACAA CCAGGAAGGACACGTCACAT
*Irs2:* Insulin receptor substrate 2	Forward Reverse	CCCATGTCCCGCCGTGAAG CTCCAGTGCCAAGGTCTGAAGG
*Pdhβ:* Pyruvate dehydrogenase E1 beta subunit	Forward Reverse	GGAGGGAATTGAATGTGAGG CATCTCGTCACTGTGGAAG
*Pgc1α*: Peroxisome proliferator-activated receptor gamma coactivator 1 alpha	Forward Reverse	AAGTGTGGAACTCTCTGGAACTG GGGTTATCTTGGTTGGCTTTATG
*Pkm2:* Pyruvate kinase M2 isoform	Forward Reverse	TGCCGTGACTCGAAATCCC GGCCAAGTTTACACGAAGGTC
*Pparα:* Peroxisome proliferator-activated receptor alpha	Forward Reverse	CCTGAACATCGAGTGTCGAATAT GGTCTTCTTCTGAATCTTGCAGCT
*Pparγ:* Peroxisome proliferator-activated receptor gamma	Forward Reverse	GCATGGTGCCTTCGCTGA TGGCATCTCTGTGTCAACCATG
*Prkag1*: Protein kinase AMP-activated non-catalytic subunit gamma 1	Forward Reverse	TCTCCGCCTTACCTGTAGTGGA GCAGGGCTTTTGTCACAGACAC

## Data Availability

The data presented in this study are available from the corresponding author upon reasonable request.

## Supplementary Materials

The following supporting information can be downloaded at: https://www.mdpi.com/article/10.3390/ijms27146530/s1.

## Abbreviations

The following abbreviations are used in this manuscript:

BAT	Brown adipose tissue
CHOL	Cholesterol
CPT1A	Carnitine palmitoyl-CoA transferase 1A
CPT2	Carnitine palmitoyl-CoA transferase 2
EE	Energy expenditure
epiWAT	Epididymal WAT
FAO	Fatty acid oxidation
FAS	Fatty acid synthase
FER	Food efficiency ratio
GOT	Glutamic oxaloacetic transaminase
GPT	Glutamic pyruvic transaminase
HSL	Hormone-sensitive lipase
HDL-C	High-density lipoprotein cholesterol
HFD	High-fat diet
MASLD	Metabolic dysfunction-associated steatotic liver disease
MCP-1	Monocyte chemoattractant protein-1
ND	Normal diet
NAFLD	Nonalcoholic fatty liver disease
non-HDL-C	Non-high-density lipoprotein cholesterol
PGC1-α	Peroxisome proliferator-activated receptor gamma coactivator 1-alpha
PPARγ	Peroxisome proliferator-activated receptor gamma
RA	Rosmarinic acid
subWAT	Subcutaneous white adipose tissue
TG	Triglyceride
TNF-α	Tumor necrosis factor-alpha
WAT	White adipose tissue
