# Supplementary figures and images for: Rosmarinic Acid Ameliorates Obesity-Associated Metabolic Disturbances and Hepatic Steatosis in Mice with High-Fat Diet-Induced Obesity
Source: Int J Mol Sci. 2026 Jul 22;27(14):6530. doi: 10.3390/ijms27146530 (PMC13411712; doi:10.3390/ijms27146530)

### Supplementary Figure S1.

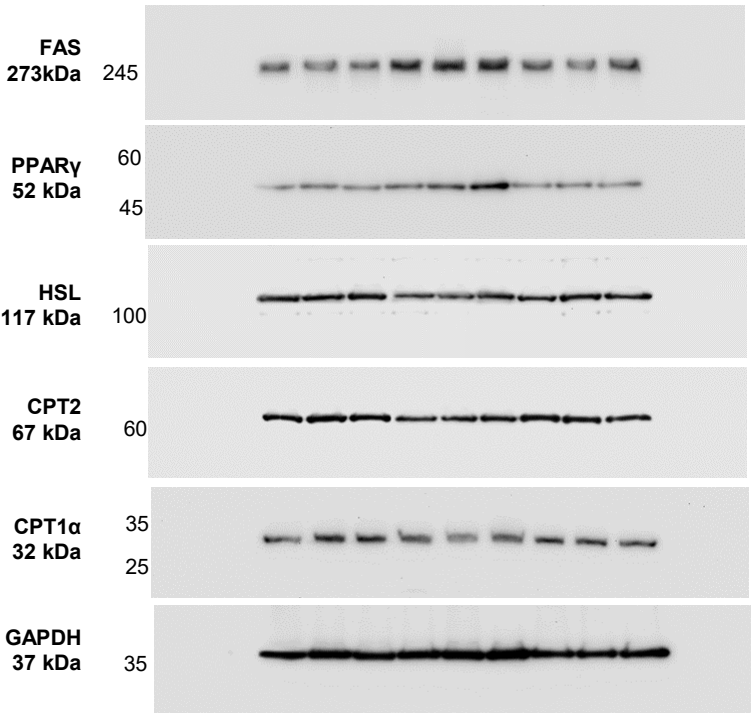

Supplementary Figure S2.

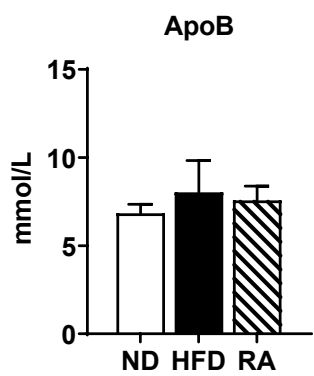

Supplementary Figure S3.

(A)

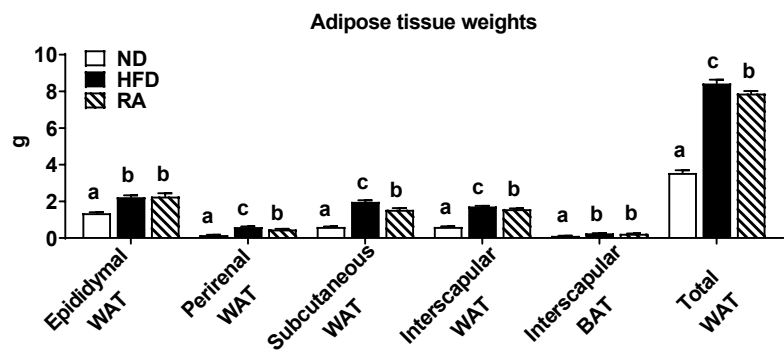

(B)

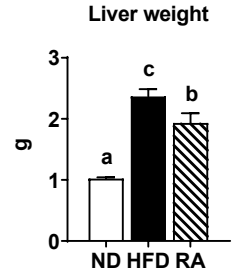

Supplement: Supplementary file 1 [file ijms-27-06530-s001.zip › ijms-4371301-supplementary.pdf]
